# Regulation of cardiac fibroblasts reprogramming into cardiomyocyte‐like cells with a cocktail of small molecule compounds

**DOI:** 10.1002/2211-5463.13811

**Published:** 2024-05-01

**Authors:** Danyang Chang, Changye Sun, Xiangqin Tian, Hongyin Liu, Yangyang Jia, Zhikun Guo

**Affiliations:** ^1^ Xinxiang Central Hospital China; ^2^ Henan Key Laboratory of Medical Tissue Regeneration Xinxiang Medical University China

**Keywords:** cardiac fibroblast, cardiomyocyte, iCMs induction, small molecule compounds

## Abstract

Myocardial infarction results in extensive cardiomyocyte apoptosis, leading to the formation of noncontractile scar tissue. Given the limited regenerative capacity of adult mammalian cardiomyocytes, direct reprogramming of cardiac fibroblasts (CFs) into cardiomyocytes represents a promising therapeutic strategy for myocardial repair, and small molecule drugs might offer a more attractive alternative to gene editing approaches in terms of safety and clinical feasibility. This study aimed to reprogram rat CFs into cardiomyocytes using a small molecular chemical mixture comprising CHIR99021, Valproic acid, Dorsomorphin, SB431542, and Forskolin. Immunofluorescence analysis revealed a significant increase in the expression of cardiomyocyte‐specific markers, including cardiac troponin T (cTnT), Connexin 43 (Cx43), α‐actinin, and Tbx5. Changes in intracellular calcium ion levels and Ca^2+^ signal transfer between adjacent cells were monitored using a calcium ion fluorescence probe. mRNA sequencing analysis demonstrated the upregulation of genes associated with cardiac morphogenesis, myocardial differentiation, and muscle fiber contraction during CF differentiation induced by the small‐molecule compounds. Conversely, the expression of fibroblast‐related genes was downregulated. These findings suggest that chemical‐induced cell fate conversion of rat CFs into cardiomyocyte‐like cells is feasible, offering a potential therapeutic solution for myocardial injury.

## Graphical abstract

Small molecule compounds present an alternative to transcription factors for modulating cellular fate through chemical signaling. This reportdescribes a series of small molecule reprogrammed of rat cardiac fibroblasts into cardiomyocyte‐like cells, exhibiting typical molecular cardiac marker,morphological characteristics, and calcium flux activity. Ultimately, these compounds hold promise in elucidating reprogramming processes and offering potential for the treatment of cardiac injury.

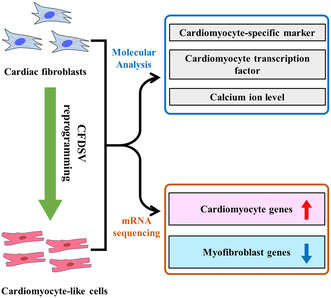

**Abbreviations**
BP: biological process
CFs: cardiac fibroblasts
CTNT: cardiac troponin T
CX43: connexin 43
DDR2: discoidin domain receptor 2
DEGs: differentially expressed genes
DMEM: Dulbecco's Modified Eagle's Medium
FACS: fluorescence‐activated cell sorting
FSP1: fibroblasts specific protein1
GO: Gene Ontology
GSEA: gene set enrichment analysis
iCMs: induced cardiomyocytes
KEGG: Kyoto Encyclopedia of Genes and Genomes
PBS: phosphate‐buffered saline
PMSF: phenylmethanesulfonyl fluoride
TBS: tris‐buffered saline

Acute myocardial infarction, resulting from coronary ischemia, is a leading cause of disability and death worldwide [1]. Myocardial infarction is often accompanied with extensive myocardial cell apoptosis and cardiac dysfunction, causing heart failure and high mortality [2, 3]. Percutaneous coronary artery ligation, bypass grafting, and diagnostic cardiac catheterization are widely employed for rapid and accurate diagnosis and treatment. However, these interventions do not permanently restore the function of damaged cardiomyocytes.

Cell therapeutics represents a promising approach for promoting myocardial repair after myocardial injury [4, 5, 6, 7, 8, 9]. Among various transplantable cells, cardiac fibroblasts (CFs) play crucial roles in maintaining heart functions, supporting tissue remodeling, and promoting the excitation and contraction of cardiac cells [10, 11, 12]. However, as a major cellular component of the cardiac interstitium, CFs can transform into myofibroblasts during heart injury, which can express extracellular matrix proteins in large quantities and lead to cardiac fibrosis [13, 14]. The direct reprogramming of CFs into cardiomyocyte‐like cells [induced cardiomyocytes (iCMs)] is a promising strategy for cardiac repair, enabling them to function as cardiomyocytes while also inhibiting myocardial fibrosis [15].

Small molecules offer a clinically applicable method for inducing cellular conversion, as demonstrated in recent studies [16, 17]. Wang *et al*. [16] employed several chemical inhibitors (SB431542, CHIR99021, Parnate, and Forskolin) to promote the transformation of mouse fibroblasts into cardiomyocytes by regulating the transcriptional activity of Oct4. Subsequently, Hou *et al*. [18] showed that mouse fibroblasts could be reprogrammed into pluripotent cells using a mixture of seven small molecules. Fu *et al*. [17] also induced mouse fibroblasts with a chemical mixture [CHIR99021, Rep Sox, Forskolin, and Valproic acid (VPA)] and transformed them into cardiomyocytes, which could express cardiomyocyte‐specific protein cTnT and exhibited electrophysiological characteristics of muscle striae and cardiomyocytes. Cao *et al*. [19] further demonstrated that human skin fibroblasts could be differentiated into cardiomyocyte‐like cells by using a small molecule combination. These studies highlight the potential of chemical methods for the regulation of differentiation of fibroblasts into cardiomyocytes. Additionally, the utilization of small molecules enables the elucidation of molecular networks and signaling pathways involved in cellular differentiation. Despite the phenotypic immaturity of cardiomyocytes, the possibility of inducing cardiomyocytes using purely chemical methods offers a promising alternative to gene therapy and has great potential for clinical application. In this study, we investigated various combinations of small molecules and identified a combination of CHIR99021, VPA, Dorsomorphin, SB431542, and Forskolin that enhanced the reprogramming of CFs into cardiomyocyte‐like cells *in vitro*. We also elucidated the roles of these chemical compounds in myocardial differentiation and the regulated genes in this process by RNA sequencing technique. These findings provide a promising avenue for myocardial repair after injury.

## Materials and methods

### Drugs and reagents

All cell culture reagents, including Dulbecco's Modified Eagle's Medium (DMEM), fetal bovine serum (FBS), and penicillin–streptomycin, were procured from Thermo Fisher Scientific (Waltham, MA, USA). Antibodies such as anti‐vimentin (ab92547), anti‐cTnT (ab8295), anti‐discoidin domain receptor 2 (DDR2) (ab221812), anti‐Tbx5 (ab137833), anti‐Nkx2‐5 (ab97355), and anti‐Ki67 (ab15580) were obtained from Abcam (Shanghai, China). Additionally, anti‐Cx43 (SAB4501175) and anti‐α‐actinin (A7811) antibodies were sourced from Sigma‐Aldrich (St. Louis, MO, USA), while anti‐fibroblast‐specific protein‐1 (FSP1) antibody (#DF6516) was acquired from Affinity (Jiangsu, China). The GSK3 inhibitor (CHIR99021) (M1692), Adenylate cyclase (AC) activator (Forskolin) (M2191), AMPK inhibitor (Dorsomorphin) (M2238), and TGF‐β inhibitor (SB431542) (M1794) were purchased from Abmole (Shanghai, China), and the histone deacetylase (HDAC) inhibitor VPA (HY‐10585) was obtained from MCE (Shanghai, China). Furthermore, the proteases inhibitor (539131) was sourced from Sigma‐Aldrich, and the Fluo‐8 calcium assay kit (1345980‐40‐6) was acquired from AAT Bioquest Inc. (Sunnyvale, CA, USA).

### CFs isolation and identification

CFs utilized in this investigation were isolated following a previously documented protocol [14]. The isolated CFs were cultured at 37 °C with 5% CO_2_, and cells at passage 3 were employed for subsequent experiments. To assess the purity of the CFs, cells were seeded at a density of 3 × 10^4^ cells per well in 48‐well plates and routinely authenticated through immunofluorescence staining with vimentin, fibroblast‐specific protein‐1 (FSP1), and discoid domain receptor 2 (DDR2), as outlined [20].

### Cell viability assay

To determine the optimal concentration of each small molecular compound, cell viability was assessed using the Cell Counting Kit‐8 (CCK8) assay (MCE). Briefly, cells were seeded into 96‐well plates at a density of 3000 cells per well in 100 μL of complete medium. After 24 h, the cells were exposed to escalating concentrations of CHIR99021 (C: 1, 2, 5, 10, 20, 40, 80, and 160 μm), Forskolin (F: 1, 2, 5, 10, 20, 40, 80 and 160 μm), Dorsomorphin (D: 0.1, 0.2, 0.5, 1, 2, 4, 8 and 16 μm), SB431542 (S: 0.5, 1, 2.5, 5, 10, 20, 40 and 60 μm), and VPA (V: 25, 50, 100, 200, 400, 600, 800, and 1000 μm) individually for an additional 48 h. After 12, 24, and 48 h, 10 μL CCK8 reagent was added to each well, and the cells were further incubated for 2 h at 37 °C. Subsequently, the optical density value (OD450) was measured using a Multiskan microplate reader (Thermo Fisher Scientific, Waltham). The assay was performed in triplicate and repeated at least three times.

### Differentiation of CFs into cardiomyocytes induced by small molecule compounds

To determine the optimal combination of small molecules, cells underwent treatment with various combinations: C, F, D, S, V, C + F, C + D, C + S, C + V, C + F + D, C + F + S, C + F + V, C + F + D + S, and C + F + D + S + V for a duration of 12 days. The cells were maintained at 37 °C with 5% CO_2_, and the growth medium was refreshed every 2 days. The most effective drug combination was identified by assessing the expression level of cTnT in each group using immunofluorescence analysis. Nuclei were stained with propidium iodide (PI). Imaging was performed using a confocal microscope (Olympus FV1000, Tokyo, Japan). For quantification, three independent experiments were processed and the data was analyzed by imagej software (Rockville, MA, USA).

### Immunofluorescence staining

The cells were rinsed with phosphate‐buffered saline (PBS), fixed in 4% paraformaldehyde, permeabilized with 0.5% Triton X‐100 in PBS, and blocked with 5% normal goat serum. Subsequently, the cells were incubated overnight with primary antibodies against Vimentin, FSP1, DDR2, cTnT, CX43, α‐actinin, Tbx5, and Nkx2‐5. After PBS washing, the cells were incubated with Alexa 488‐labeled and Cy3‐labeled goat anti‐rabbit or anti‐mouse secondary antibodies for 2 h at room temperature. Nuclei were counterstained with 4′,6‐diamidino‐2‐phenylindole (DAPI) for 15 min. The fluorescence images were captured using a fluorescence microscope. The percentage of positive cells (positive rate = number of positive cells/total number of nuclei × 100%) for Vimentin, FSP1, DDR2, and cTnT, respectively, was determined in five randomly selected fields in each group. All experiments were conducted in triplicate.

### Flow cytometry

Cardiac fibroblasts were initially cultured in a 75 cm^2^ culture dish and exposed to the optimal concentration of CFDSV for flow cytometry analysis after 24 h. A total of 500 μL of cell suspension containing 50 000–100 000 cells were collected and filtered through a 100‐mesh strainer into a 1.5 mL centrifuge tube. These cells were then fixed with 4% paraformaldehyde for 15 min at room temperature and incubated with 0.3% Triton X‐100 in PBS for another 15 min. After centrifugation at 400–600 ***g*** for 5 min at room temperature to discard the supernatant, the cells were blocked with 3% goat serum for 1 h. Subsequently, the cells were incubated with a mouse monoclonal anti‐cTnT antibody at a 1 : 500 dilution in PBS buffer for 90 min at room temperature. Following three washes with PBS, the cells were labeled with a goat anti‐mouse Alexa 488 secondary antibody for 1 h at room temperature in the dark. The cells were then resuspended in 500 μL of PBS and analyzed using a flow cytometer BD FACS ARIA II (BD Bioscience, Franklin Lakes, NJ, USA).

### Cell index assay

The cell index (CI) was utilized to monitor cell proliferation dynamics in the form of a growth curve using an ECR real‐time analysis system (ACEA Biosciences Lnc., San Diego, CA, USA). Initially, 50 μL of DMEM was placed in the E‐plate 96 and incubated at 37 °C with 5% CO_2_ to measure the background. Subsequently, 100 μL of cell suspension (3000 cells per well) in 10% FBS DMEM was added to the plate and left at room temperature for 30 min to ensure cell suspension at the bottom of the plate. After 24 h of incubation, the medium was replaced with 100 μL of serum‐free medium for cell starvation, and the CI was recorded every 5 min for 24 h. The cells were then stimulated with CFDSV and monitored hourly for 48 h, every 4 h from 48 to 102 h, and every 10 h from 102 to 402 h to obtain the CI over 12 days. Each independent experiment was conducted in triplicate, and the interval slope was automatically calculated by the RTCA software to assess the rate of CI change [21].

### RNA sequencing analysis

Total RNA of the control group and CFDSV group were isolated using a TRIzol reagent (Thermo Fisher Scientific, Shanghai, China). RNA‐seq and bioinformatics analysis were performed at the Beijing Genome Institute (Shenzhen, China) as previously described [22]. Differentially expressed genes (DEGs) between the two samples were identified by selecting significantly changed genes with *Q*‐value < 0.01 and log_2_(fold change) ≥ 2 or ≤ −2 for further analysis. Gene Ontology (GO) enrichment analysis, Kyoto Encyclopedia of Genes and Genomes (KEGG) analysis, and gene set enrichment analysis (GSEA) were conducted using the DAVID bioinformatics resources portal and clusterprofiler package in r (3.6) as described previously [23, 24].

### Quantitative real‐time PCR analysis

Primers for the target genes were designed using oligo 6.0 software (Molecular Biology Insights, Inc., Cascade, CO, USA) and synthesized by Sangon Biotech Company (Shanghai, China). The primer sequences for the target genes are presented in Table S1. Total RNA was isolated with TRIzol reagent (Thermo Fisher Scientific, Shanghai) and reverse‐transcribed into cDNA using cDNA Synthesis Super Mix (Thermo Fisher Scientific, Shanghai). qRT‐PCR was performed using the Light‐Cycler 480 II PCR detection system (Roche, Basel, Switzerland) and SYBR Premix Ex TaqTM II (TAKARA Bio Inc., Dalian, China) according to the manufacturer's instructions. The experiment was repeated three times to ensure the robustness and reliability of the results and mRNA levels were normalized by comparison to *Gapdh* mRNA.

### Western blot analysis

Cells from the control and CFDSV groups were washed in PBS, scraped in lysis buffer (Beyotime, Beijing, China) containing protease inhibitor phenylmethanesulfonyl fluoride (PMSF), and incubated on ice for 30 min. Total proteins were separated on 10% SDS/PAGE gels, transferred onto polyvinylidene difluoride membranes, and blocked with 5% nonfat milk in TBST buffer. The membranes were then probed with primary antibodies (against cTnT, α‐actinin, Cx43, and Tbx5) overnight at 4 °C. After incubation with HRP‐conjugated goat anti‐rabbit or anti‐mouse IgG, blots were probed for β‐tubulin to normalize protein content. Protein bands were visualized using an ECL system.

### Measurements of intracellular Ca^2+^ dynamics

To investigate the impact of CFDSV on intracellular Ca^2+^, we employed the fluorescence dye Fluo‐8‐AM to measure Ca^2+^ levels according to the standard protocol. Briefly, cells were seeded at 40 000 cells/100 μL/well in a 96‐well black wall/clear bottom costar plate. Small molecule medium CFDSV was added to stimulate the cells for 16 days, the growth medium was removed, and the cells were incubated with Fluo‐8 AM at a concentration of 4 μm in a 37 °C, 5% CO_2_ incubator for 1 h to load the dye. After being washed twice with HHBS, the cells were imaged with a fluorescence microscope (Olympus xCellence) using FITC channel. The images obtained were digitized by imagej software, and the fluorescent intensity is shown in a line plot.

### Statistical analysis

Statistical analyses were performed using the software GRAPHPAD PRISM 7.0 (GraphPad Software Inc., San Diego, CA, USA), with data expressed as means ± SEM. Differences between groups were analyzed using Student's *t*‐test or one‐way ANOVA, with statistical significance set at *P* < 0.05 (*p < 0.05, **p < 0.01, and ***p < 0.001).

## Results

### Purity identification of CFs

**Fig. 1:**
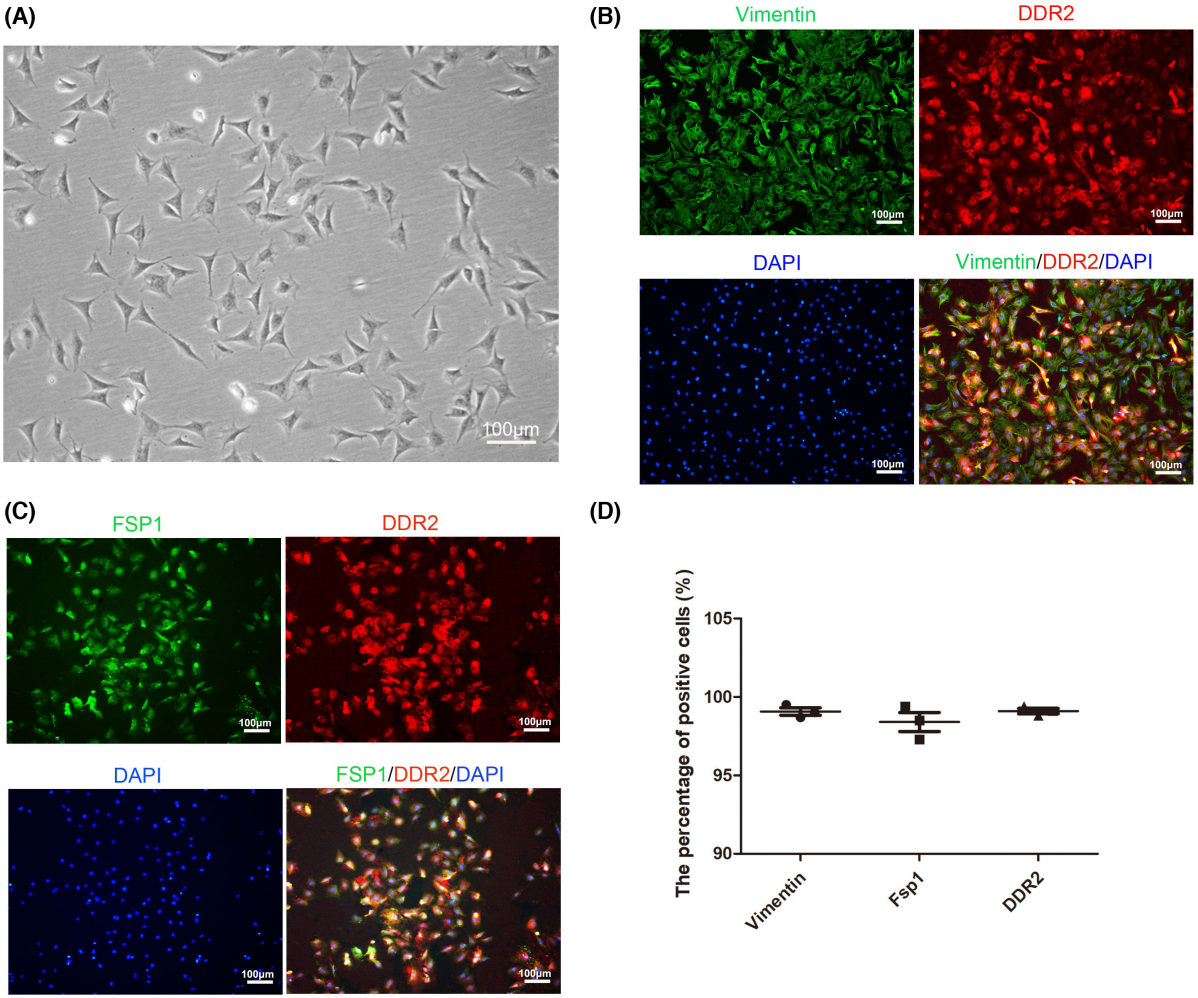
Morphology and identification of cardiac fibroblasts (CFs) with Vimentin, discoidin domain receptor 2 (DDR2), and fibroblasts specific protein1 (FSP1). (A) Morphology of CFs by phase‐contrast imaging. (B) Colocalization of Vimentin and DDR2 on passage 3 of CF detected by immunofluorescence assays. (C) Colocalization of FSP1 and DDR2 on passage 3 of CF detected by immunofluorescence assays. Scale bars: 100 μm. (D) Statistics of positive expression rates of Vimentin, FSP1, and DDR2. The difference was assayed using one‐way ANOVA with the Tukey–Kramer posttest. Data are presented as the mean ± SEM; *n* = 3.

Cardiac fibroblasts exhibited typical spindle‐ or triangle‐like shapes with flat cytoplasm under a light microscope (Fig. 1A). To verify the purity of fibroblasts, we utilized a combination of markers including Vimentin, FSP1, and DDR2, commonly associated with CFs. Immunofluorescence analysis revealed co‐expression of these markers in the cytoplasm of CFs (Fig. 1B,C), with expression rates of Vimentin, DDR2, and FSP1 at 99.07%, 99.1%, and 99.4%, respectively (Fig. 1D). Based on this evidence, we interchangeably used CFs and these markers throughout the study.

### Screening of small molecule compounds for cardiomyocyte reprogramming

**Fig. 2:**
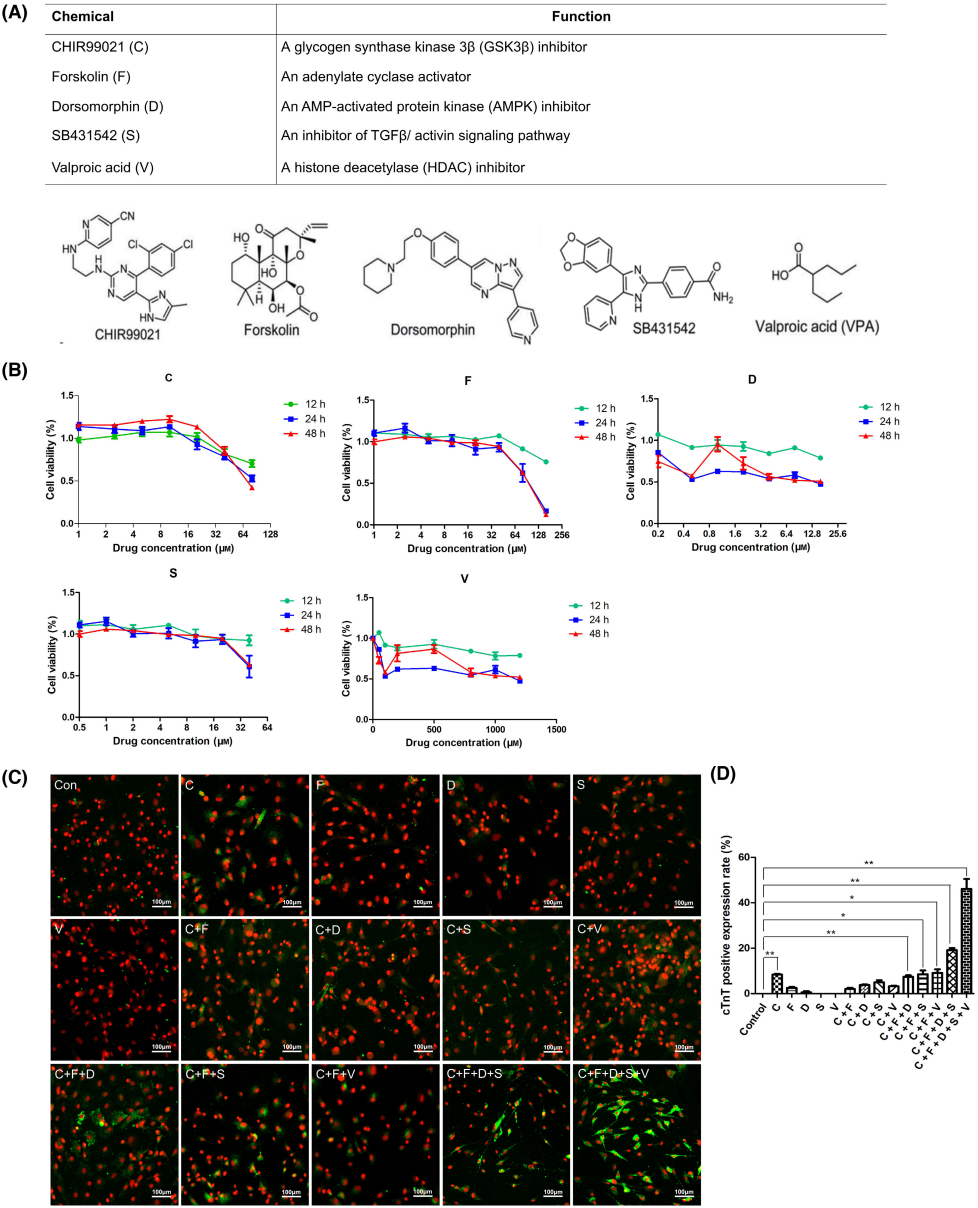
Screening of the optimal concentration and combinations of small molecule compounds. (A) Functions and structures of five small molecules compounds. (B) Effect of five small molecule compounds on cell viability of fibroblasts in different concentration ranges. (C) Cardiac fibroblasts (CFs) treated with different combinations of chemical cocktail were immunostained with antibodies against cardiac‐specific marker cardiac troponin T. Scale bars: 100 μm. (D) Quantification of cTnT positive rate at Day 10 treated with different chemical cocktail were measured and statistically analyzed. The difference was assayed using one‐way ANOVA with the Tukey–Kramer posttest. Data are presented as the mean ± SEM; *n* = 3. *P < 0.05, **P < 0.01 versus relevant control.

Previous studies have identified numerous small molecules that play roles in the reprogramming process, targeting pathways such as Wnt, TGF‐β, GSK3, BMP, and activin signaling pathways [19, 25]. Our screening process indicated that a combination of CHIR99021, Forskolin, Dorsomorphin, SB431542, and VPA effectively facilitated the generation of cardiac cells from fibroblasts (Fig. 2A). To determine the optimal concentration for action, we investigated the impact of varying concentrations of these small molecules on cell viability. The primary concentrations identified were CHIR99021 (8–10 μm), Forskolin (5–16 μm), Dorsomorphin (0.5–1.6 μm), SB431542 (2–8 μm), and VPA (400–600 μm) (Fig. 2B). Based on these results and existing literature, we established the optimal concentration as CHIR99021 (10 μm), Forskolin (10 μm), Dorsomorphin (1 μm), SB431542 (5 μm), and VPA (500 μm). Among the screened small molecules, the GSK3 inhibitor (CHIR99021) significantly enhanced the expression of the cardiomyocyte marker cTnT in CFs (Fig. 2C,D). Conversely, the other tested compounds, including AMPK inhibitor (Dorsomorphin), Adenylate cyclase activator (Forskolin), TGF‐β inhibitor (SB431542), and HDAC inhibitor VPA each did not enhance cTnT expression (Fig. 2C,D). We also screened the combination of two small molecules to achieve reprogramming efficiency. However, the reprogramming efficiency was not improved compared with single drug treatment. The reprogramming efficiency was enhanced by the combination of CHIR99021 and Forskolin with any of the remaining three compounds. In contrast, the reprogramming efficiency was further enhanced by the combination of CHIR99021, Forskolin, Dorsomorphin, and SB431542, and the combination of these five small molecule compounds acted together to achieve the highest reprogramming efficiency (Fig. 2C,D). This screening procedure showed that a mixture of CHIR99021, Forskolin, Dorsomorphin, SB431542, and VPA serves as an effective chemical cocktail for generating cardiac cells from fibroblasts, and CHIR99021 is essential molecules for reprogramming.

### Small molecule combinations increase the proportion of induced cardiomyocyte‐like cells

**Fig. 3:**
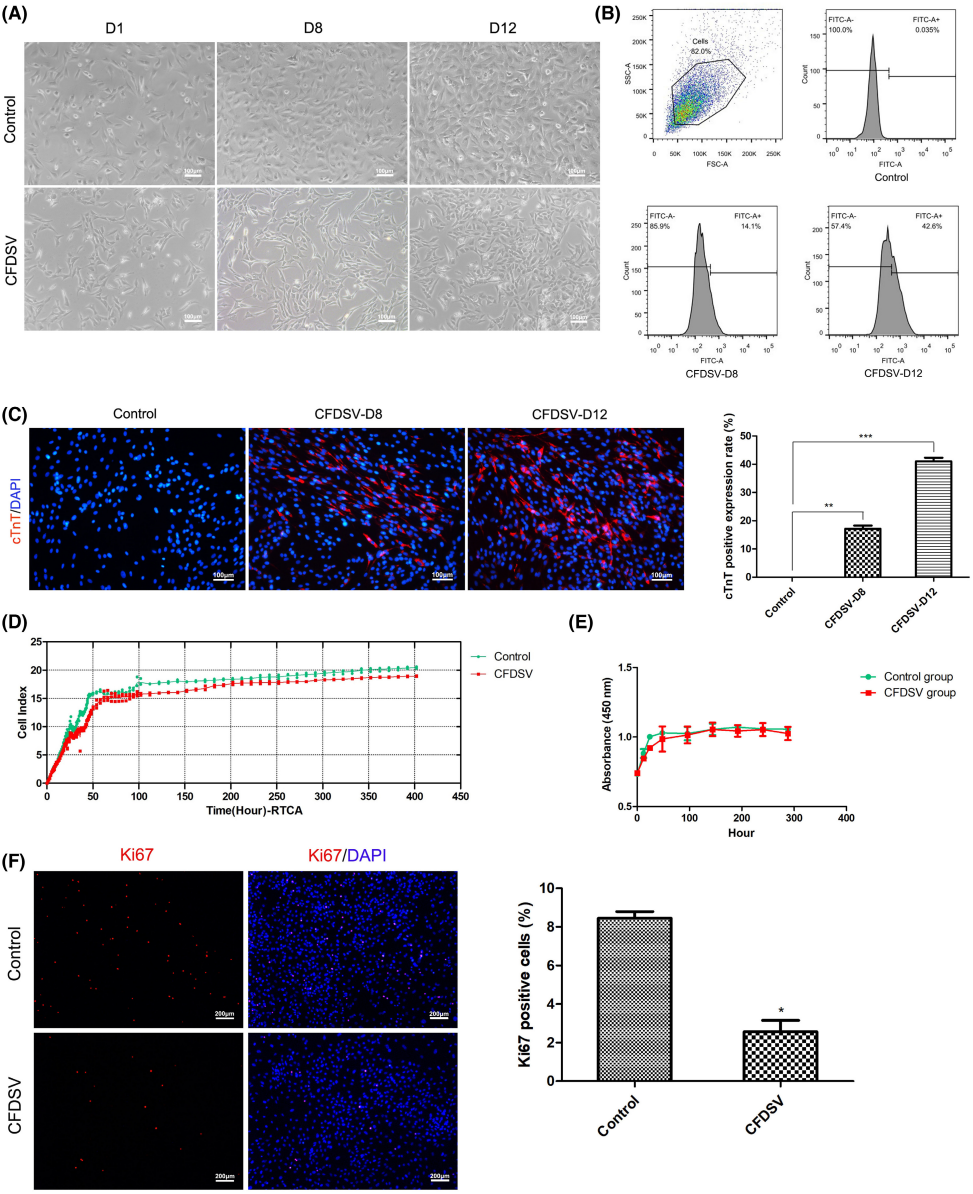
CFDSV enhance cardiac reprogramming in rat cardiac fibroblasts (CFs) and inhibits cell proliferation. (A) Morphological characteristics of cells treated with CFDSV. Scale bars: 100 μm. (B) The positive rate of cTnT in different groups was analyzed by flow cytometry. (C) Representative immunocytochemistry images (left) and quantification (right) of cTnT^+^ cells 8 and 12 days after CFDSV treatment of CFs. Scale bars: 100 μm. (D) Changes in cell index (CI) over the course of cardiac reprogramming. Horizontal axis shows the time (h); vertical axis shows the CI. (E) Cell growth curve changes during cardiac reprogramming. Horizontal axis shows the time (h); vertical axis shows the OD value. (F) Representative immunocytochemistry images (Left) and quantification (Right) of Ki67^+^ cells. Scale bars: 200 μm. The difference was assayed using one‐way ANOVA with the Tukey–Kramer posttest. Data are presented as the mean ± SEM; *n* = 3. *P < 0.05, **P < 0.01, ***P < 0.001 versus the relevant control.

Treatment with small molecule compounds induced significant alterations in cellular morphology, with cells exhibiting elongated, spindle‐shaped appearances in the CFDSV‐treated group compared with the control group (Fig. 3A). Flow cytometry studies demonstrated that rat CFs treated with a combination of five small molecules exhibited increased cTnT expression compared with untreated cells. Additionally, treatment with CFDSV led to the induction of 14.1% cTnT^+^ cells on Day 8 postinduction and 42.6% cTnT^+^ cells on Day 12 postinduction in rat CFs (Fig. 3B). Similarly, immunofluorescence staining for cTnT (Fig. 3C) demonstrated that CFDSV induced 17.1 ± 1.9% cTnT^+^ cells on Day 8 postinduction, while reach up to 41.1 ± 2.2% cTnT^+^ cells on Day 12. These results collectively highlight the effectiveness of CFDSV in promoting the reprogramming of CFs.

### Small molecule combinations inhibit cell proliferation

Considering that CFDSV can increase the efficiency of cardiac reprogramming, we then investigated whether the effects of CFDSV might be related to cell proliferation. To test these hypotheses, immunofluorescence staining was employed to quantify proliferating iCMs marked by cTnT expression and total cell numbers (DAPI‐positive cells) at multiple time points over a 12‐day period (Fig. 3C). The fluorescence staining revealed a significant increase in the number of cTnT^+^ cells, while there was no significant increase in the number of nuclei. This assay showed that CFDSV enhanced cardiac reprogramming efficiency by increasing the absolute number of cTnT^+^ iCMs. Results of CI analysis showed that CFDSV inhibiting cell proliferation and decreasing the absolute number of total cells and thus resulting in the higher ratio of iCMs to total cells (Fig. 3D). Notably, the cell proliferation curve demonstrated a significant inhibition of CFs proliferation for a transient period of 48 h following CFDSV treatment (Fig. 3E). Additionally, Ki67 staining was performed (Fig. 3F) to further confirm that CFDSV‐induced iCMs without stimulating iCMs proliferation. These collective findings suggest that CFDSV promotes cardiac reprogramming by increasing the absolute number of iCMs while inhibiting cell proliferation, thereby enhancing the conversion ratio.

### GO enrichment analysis

**Fig. 4:**
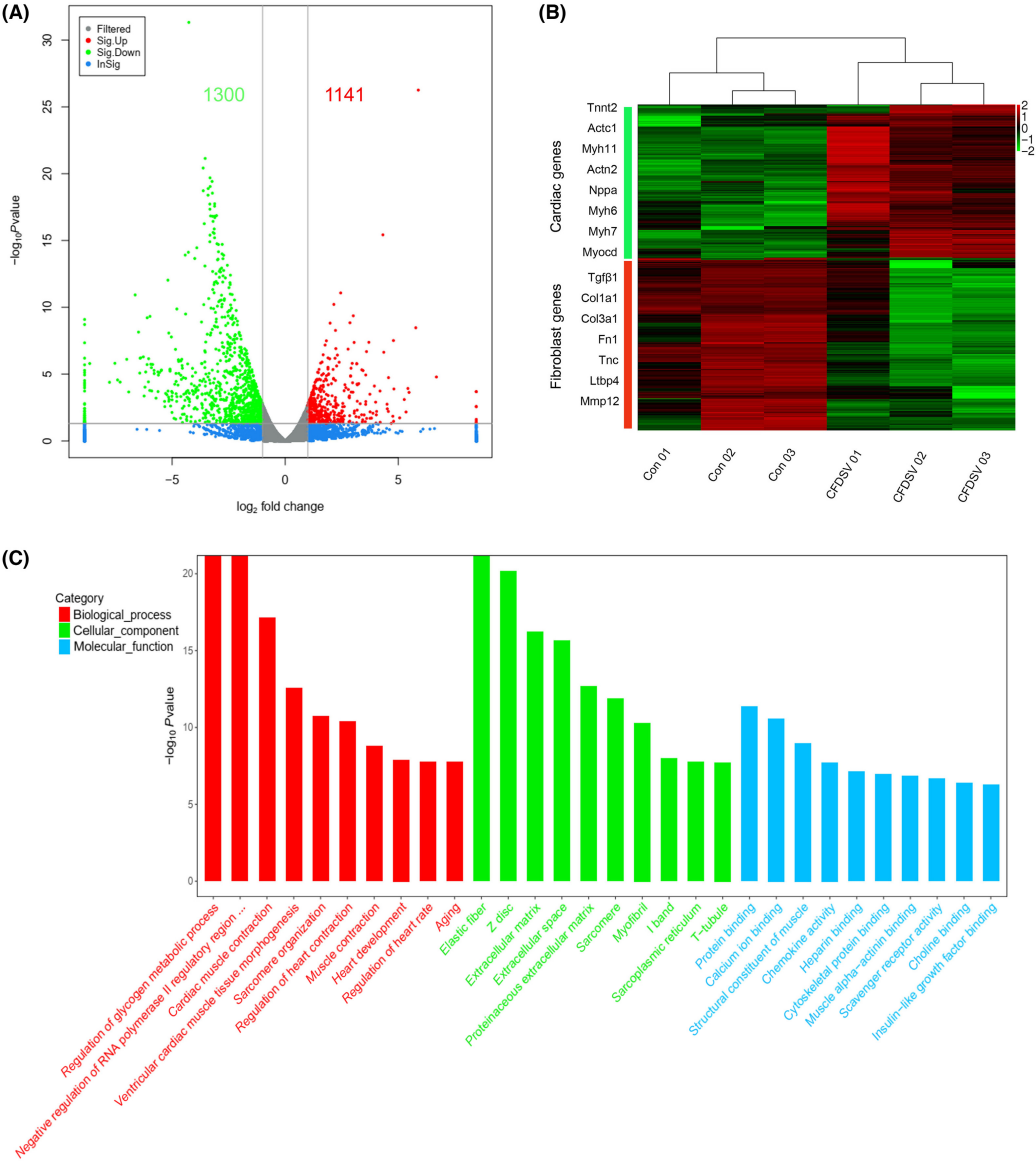
Gene set enrichment analysis. (A) Volcano map of differentially expressed genes (DEGs) between CFDSV and control treatment on cardiac fibroblasts (CFs) 4 weeks postinduction. (B) Heatmap image of microarray data illustrating DEGs between control and CFDSV group (4 weeks postinduction.). The scale extends from −2 to +2 in log_2_ scale. (C) Enriched Gene Ontology (GO) terms for differentially regulated genes in CFs treated with CFDSV for 4 weeks. The horizontal axis shows the biological function annotation category; the vertical axis represents the size of the *Q*‐value (−log_10_(*Q*‐value)), which is shown in the bar chart.

To assess the quality of reprogrammed cells, RNA‐seq was conducted on cells after 4 weeks of CFDSV‐induced reprogramming. The gene expression profiles in iCMs exhibited improvements compared with CFs, as evidenced by the expression levels of key cardiac and fibroblast genes in iCMs. Utilizing specific criteria (log_2_ ratio ≥ 1 or ≤ −1, *q*‐value ≤ 0.01, and FDR < 0.01), we identified 2441 DEGs, with 1141 upregulated and 1300 downregulated genes (Fig. 4A,B).

Furthermore, GO classification and functional enrichment analysis of the differentially regulated genes between the control and CFDSV groups revealed enrichment in biological processes (BPs) such as cardiac muscle contraction, cardiac muscle tissue morphogenesis, sarcomere organization, regulation of heart contraction, and heart development, all of which are crucial in cardiomyocyte formation‐related processes. Molecular functional changes primarily involved protein binding, calcium ion binding, structural constituent muscle, muscle α‐actinin binding, and more. Changes in cellular components were associated with modifications in elastic fiber, extracellular matrix, sarcomere structure, myofibril composition, and other components, closely linked to myocardial cell components and tissue fibrosis (Fig. 4C).

**Fig. 5:**
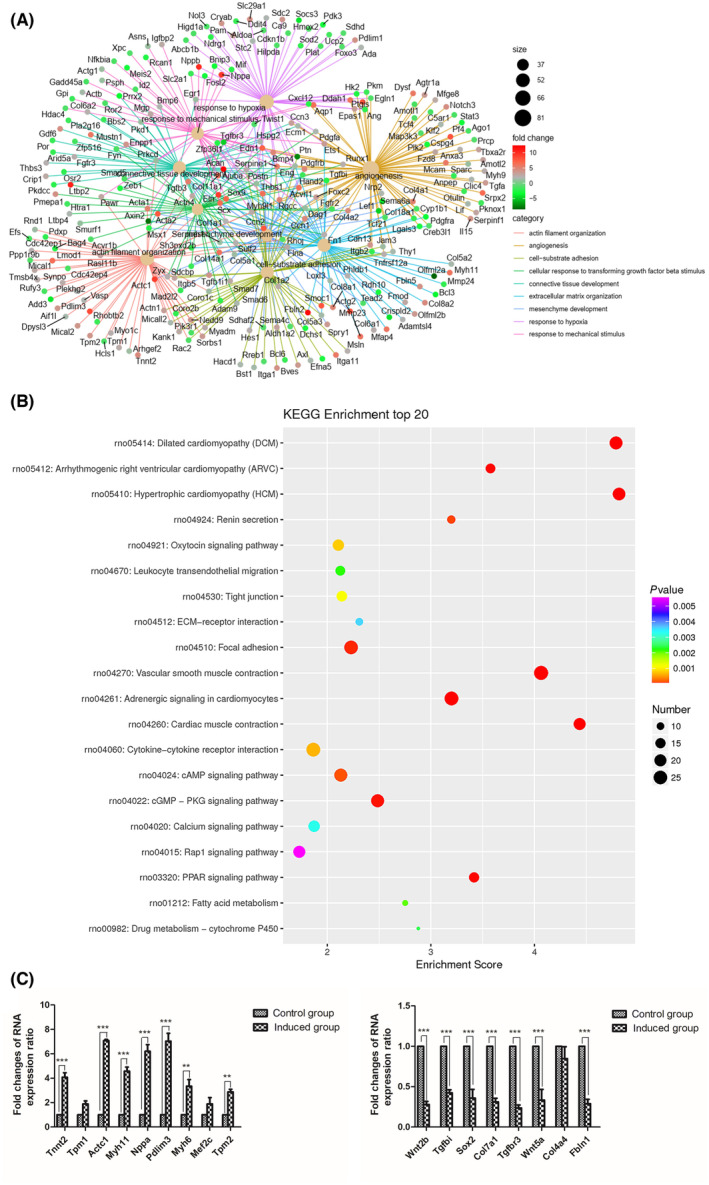
Enriched functions and genes related to cardiac reprogramming. (A) The Gene‐Concept Network of differentially expressed genes (DEGs) functional enrichment in biological process (BP). (B) Kyoto Encyclopedia of Genes and Genomes (KEGG) enrichment analysis of DEGs between CFDSV and control treatment on 4‐week cultured cardiac fibroblasts. (C) Relative mRNA level of cardiomyocyte‐specific gene and fibroblast‐related gene expression in cells treated with CFDSV for 4 weeks, determined by qPCR. *Gapdh* expression was used as the normalization control. The difference was assayed using one‐way ANOVA with the Tukey–Kramer posttest. Data are presented as the mean ± SEM; *n* = 3. **P < 0.01, ***P < 0.001 versus relevant control.

By analyzing the top 10 BPs enriched in genes, we observed that the small molecules enhanced the expression of cardiac genes while suppressing fibroblast gene expression. Notably, functionally significant cardiac genes such as *Actc1* (cardiac muscle actin), *Tnnt2* (cardiac troponin T type 2), *Actn2* (actinin), *S100a6* (S100 calcium‐binding protein A6), *Tpm2* (tropomyosin 2), *Nppa* (natriuretic peptide A), and *Myl9* (myosin light polypeptide 19), among others, were significantly upregulated. Conversely, the expression of fibroblast marker genes, including *Col1a1* (collagen 1a1), *Col3a1* (collagen 3a1), *Fn1* (fibronectin 1), and *Tnc* (tenascin C), was downregulated after 4 weeks of reprogramming (Fig. 5A). Differentially expressed genes involved in pathways such as cardiac muscle contraction, cGMP‐PKG signaling, cAMP signaling, calcium signaling, and Rap1 signaling were identified and displayed in KEGG pathways (Fig. 5B).

Furthermore, qRT‐PCR analysis demonstrated significant upregulation of genes related to myocardial development, including *Tnnt2*, *Actc1*, *Myh6*, *Mef2c*, *Pdlim3*, *Myh11*, *Nppa*, and *Tpm2*, while genes associated with fibrosis such as *Wnt2b*, *Tgfbi*, *Col7a1*, *Sox2*, and *Fbln1* were significantly downregulated (Fig. 5C). The consistency in expression trends between qRT‐PCR and RNA sequencing data underscores the reliability of the transcriptomic data utilized in this study (Tables S2 and S3).

### Enrichment analysis for DEGs

**Fig. 6:**
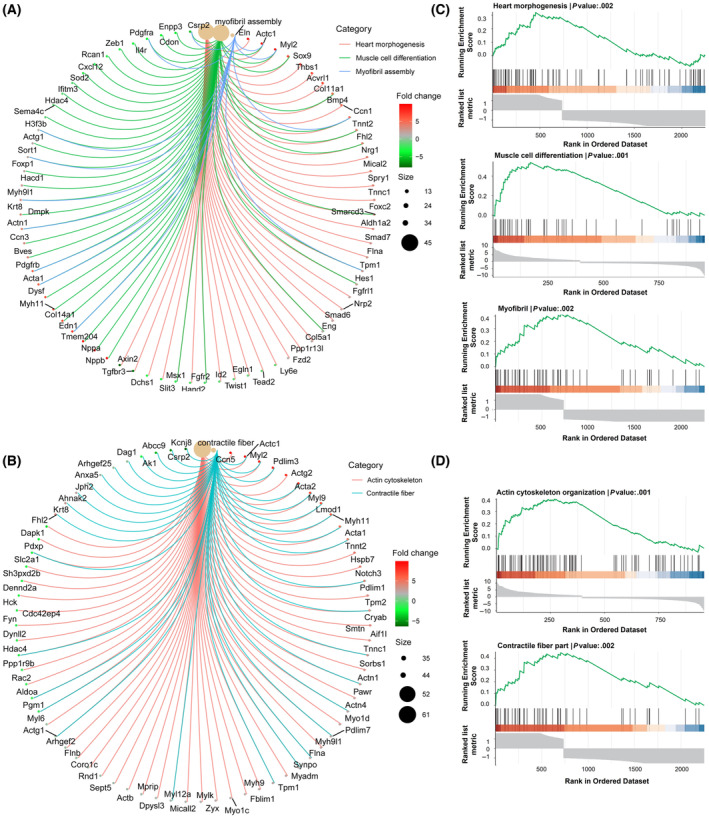
Enriched functions and relevant genes in cellular component cluster. (A) Display of genes related to heart morphogenesis, muscle cell differentiation, and myofibril assembly. (B) Display of genes related to Actin cytoskeleton and contractile fiber. (C) Gene set enrichment analysis (GSEA) plots of heart morphogenesis, muscle cell differentiation, and myofibril assembly. (D) GSEA plots of Actin cytoskeleton and contractile fiber.

The GO and GSEA results identified the DEGs enriched in related BPs and their regulatory trends. The upregulated genes, such as *Actc1*, *Acta1*, *Myl2*, *Myh11*, *Sox9*, *Tpm1*, and *Myo1d*, were notably enriched in BPs related to cardiac morphogenesis, muscle cell differentiation, myofibrillar assembly, actin cytoskeleton organization, and contractile fiber formation (Fig. 6A,B). These results strongly suggest that CFDSV significantly enhances the differentiation of cells into cardiomyocytes. Subsequently, GSEA was employed to assess the alteration in biological function defined by a specific gene set. The enrichment of processes such as heart morphogenesis, muscle cell differentiation, myofibril organization, actin cytoskeleton arrangement, and contractile fiber formation were upregulated, which indicates that CFDSV compounds enhance myocardial reprogramming in CFs (Fig. 6C,D).

### Small‐molecule compounds enhance cardiac reprogramming in a time‐dependent manner

**Fig. 7:**
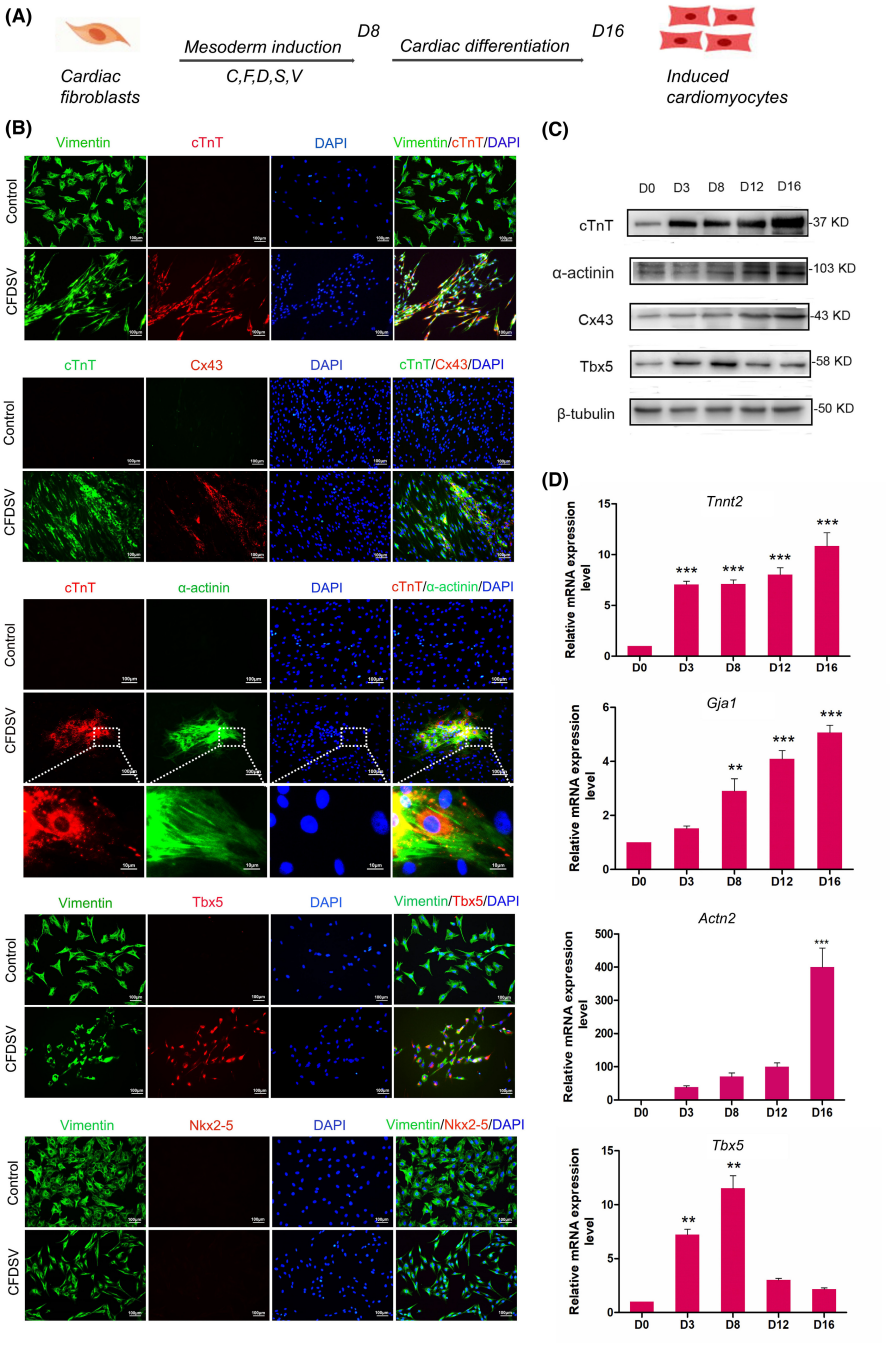
Conversion of cardiac fibroblasts (CFs) into cardiomyocytes by compound combination CFDSV. (A) Scheme of stepwise conversion of CFs to cardiomyocytes. (B) Representative immunocytochemistry images of cTnT^+^ cells, Cx43^+^ cells, α‐actinin^+^ cells, Tbx5^+^ cells, and Nkx2‐5^+^ cells after CFDSV treatment of human CFs. Scale bars: 100 μm. The high magnification picture shows well‐organized sarcomere structure. Scale bar: 10 μm. (C) The cTnT, Tbx5, α‐actinin, and Cx43 protein expression level in CFs over time for groups treated with CFDSV were determined by western blot analysis. (D) Statistical analysis of *Tnnt2*, *Tbx5*, *Gja1*, and *Actn2* mRNA expression detected by RT‐PCR during cardiac reprogramming. The difference was assayed using one‐way ANOVA with the Tukey–Kramer posttest. Data are presented as the mean ± SEM; *n* = 3. **P < 0.01, ***P < 0.001 versus relevant control.

Cardiac fibroblasts were treated with CFDSV for 8 and 16 days to determine the effect of CFDSV on the reprograming of CFs into cardiomyocyte‐like cells (Fig. 7A). Immunofluorescence staining revealed a significant upregulation of cardiomyocyte‐specific markers, including α‐actinin, cTnT, Tbx5, Cx43, and Nkx2‐5 in cells treated with the small‐molecule compound CFDSV. A substantial increase in cTnT‐expressing cells was observed postreprogramming with CFDSV. Additionally, CFDSV treatment led to the expression of α‐actinin^+^ iCMs that assembled sarcomeres compared with the control group. Formation of gap‐junction protein Gja1 channels was also observed following CFDSV treatment. Moreover, endogenous Tbx5 expression was upregulated in iCMs. However, the expression of the cardiac progenitor cell marker Nkx2‐5 was not detected during reprogramming (Fig. 7B).

Immunoblotting analysis of cardiac‐related proteins during reprogramming was also shown that the cardiomyocyte‐specific markers, α‐actinin, cTnT, Cx43, and Tbx5 were significantly upregulated in a time‐dependent manner in cells treated with small‐molecule compounds CFDSV. The temporal dynamics of Tbx5 expression during reprogramming were analyzed. The expression of Tbx5 commenced on Day 3 and subsequently exhibited a gradual increase, culminating on Day 8 before gradually declining. This provides further evidence supporting the role of Tbx5 as an early cardiac transcription factor involved in initiating the expression of genes crucial for cardiac development. A similar pattern can be observed in the temporal expression of cTnT and Cx43 throughout cellular reprogramming (Fig. 7C).

These findings were further validated by qRT‐PCR analysis, showing an increased expression of genes associated with cardiomyocyte structure (*Tnnt2*, *Actn2*, and *Gja1*), and differentiation (*Tbx5*) in a time‐dependent manner in CFDSV‐treated cells (Fig. 7D).

### Induced cardiomyocytes exhibit spontaneous Ca^2+^ flux

**Fig. 8:**
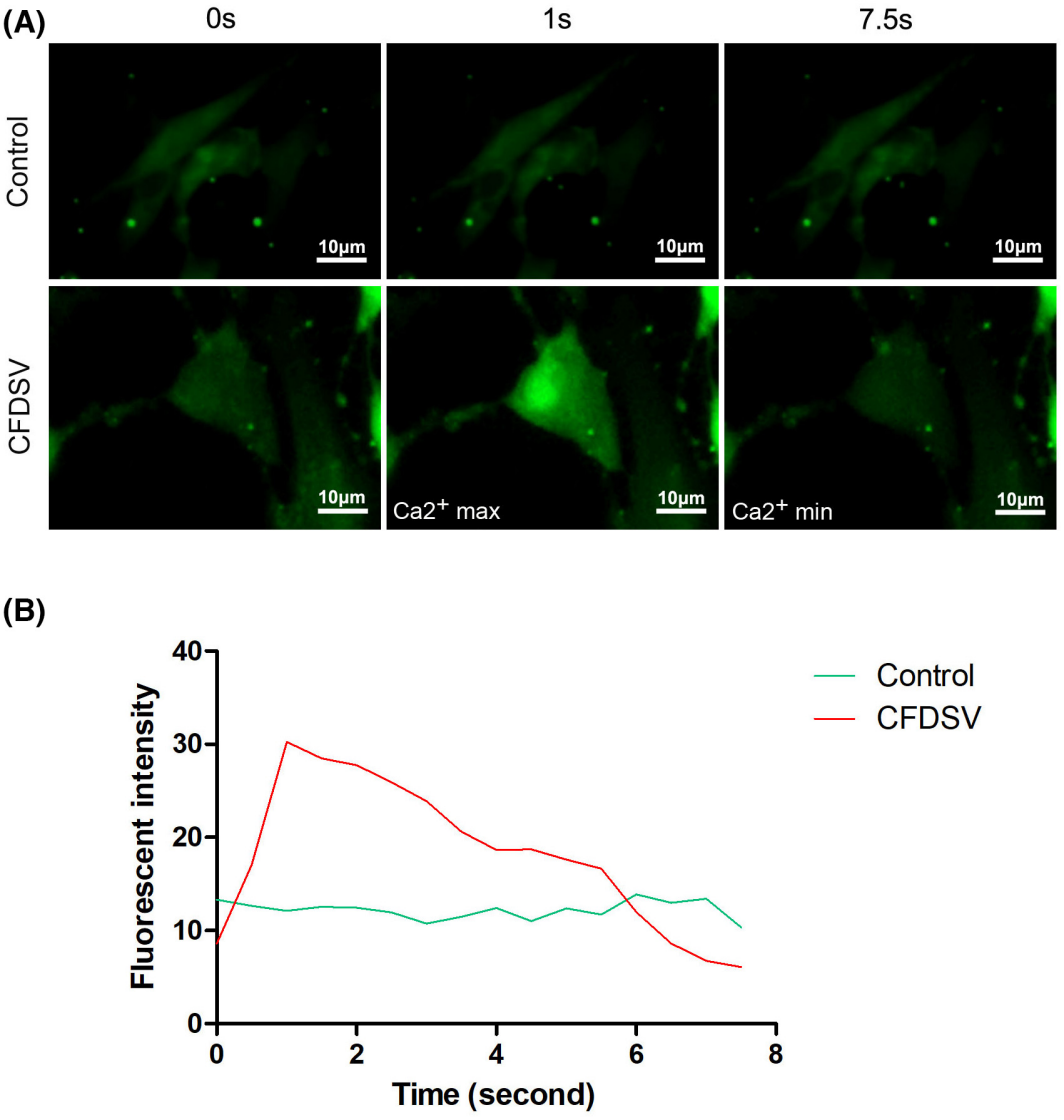
Induced cardiomyocytes exhibit spontaneous Ca^2+^ flux. (A) Spontaneous Ca^2+^ oscillation observed at the Ca^2+^ max and min of the CF‐derived induced cardiomyocytes (iCMs). Scale bars: 10 μm. Fluorescent images correspond to the Movies S1–S3. (B) Time course of fluo‐8 intensity change in CF‐derived iCMs showed spontaneous Ca^2+^ oscillation. The fluorescence intensity was analyzed at 16 arbitrary points. Each line shows the raw fluorescence intensity data at each point.

To determine whether CFDSV‐induced iCMs have the functional properties of cardiomyocytes, we conducted an analysis of intracellular Ca^2+^ flux, a critical regulator of cardiomyocyte contraction. This analysis involved monitoring fluorescence intensity in iCMs after a 4‐week culture period. The CF‐iCMs displayed spontaneous Ca^2+^ oscillations, and the fluorescence intensities of CF‐iCMs treated with CFDSV were notably higher than those of the control group (Fig. 8A,B). Fluorescent images correspond to the Movies S1 and S2. Fluo‐8 fluorescence rapidly increased in the patched cell, followed by a fluorescence increase in adjacent cells in contact with the patched cell just a few seconds (Movie S3). These results suggest that the reprogramming of fibroblasts into iCMs led to the activation of ion channels and the intercellular transport of calcium ions, thereby establishing a fundamental basis for cellular functionality.

## Discussion

The generation of functional cardiovascular cells plays a pivotal role in cardiovascular disease research. A promising avenue in regenerative medicine for heart disease treatment involves the direct reprogramming of iCMs from fibroblasts and other somatic cells. This innovative technique offers a novel perspective on cardiac repair, converting the fibrotic process into a reparative mechanism that yields functional cardiomyocytes, thereby reversing cardiac damage.

Numerous studies have explored diverse combinations of transcription factors such as Gata4, Mefc2, and Tbx5, along with growth factors, kinases, small molecules, and microRNAs to enhance myocardial reprogramming *in vitro* [25, 26, 27, 28]. The utilization of small molecules has facilitated *in vitro* reprogramming to a certain extent, offering chemical tools for investigating potential reprogramming mechanisms. Several small molecule combinations, such as CRFVPTZ (comprising CHIR99021, RepSox, Forskolin, VPA, Parnate, TTNPB, and DZnep), have been identified as catalysts for reprogramming [17, 18, 29]. The advantage of small molecule reprogramming lies in its simplicity and controllability, enabling direct gene expression activation from the somatic genome or compensating for its activity without gene insertion and oncogenic risks [30, 31].

The utilization of small molecules in reprogramming can effectively target diverse signaling pathways implicated in heart development and function, including Wnt, TGF‐β, and BMP. The presentation focuses on these compounds, primarily in relation to their role in myocardial differentiation, and includes references to relevant literature. For example, the small molecule CHIR99021 can inhibit the GSK signaling pathway, leading to initial reprogramming and induction of cardiac mesoderm [32]. Similarly, SB431542 inhibits the TGF‐β signaling pathway, thereby inhibiting induced transcription, gene expression, apoptosis, and cell proliferation [25]. Forskolin is a potent activator of adenylate cyclase, which promotes the formation of intracellular cAMP, thereby inducing cellular differentiation and autophagy. [33]. Dorsomorphin, as an inhibitor of BMP signaling pathway, can induce Smad activation and promote the generation of cardiomyocytes [34]. The inhibition of HDAC by VPA enables the regulation of cellular proliferation, apoptosis, and differentiation [35].

In our study, we demonstrated that a combination of five small molecule compounds (CHIR99021, Forskolin, Dorsomorphin, SB431542, and VPA) promptly and effectively iCMs‐like cells from CFs *in vitro*. The cardiac reprogramming efficiency reached approximately 40% after 2 weeks, with the iCMs displaying well‐defined sarcomere structures and spontaneous Ca^2+^ oscillations after 4 weeks. These iCMs exhibited a global gene expression profile similar to that of cardiomyocytes. While further refinements and characterizations of the reprogramming process are imperative, the successful conversion of a considerable number of fibroblasts into functional cardiomyocyte‐like cells presents a promising advancement. Although it is conceivable that higher reprogramming efficiencies could be achieved either in conjunction with transcription factors, or transcription factors alone, the small molecule approach offers a significant advantage over the transgenic approach due to its ease of combination, and controllability [16]. Furthermore, small molecule‐based reprogramming reduces the risk of tumor formation, enables autologous cell transplantation, and targets multiple signaling pathways.

Our findings demonstrated a significant enhancement in the efficiency of cardiac reprogramming with CFDSV. In order to verify the reliability of this result, we studied the effect of CFDSV on cell proliferation by Ki67 staining, and the results confirmed that the conversion rate of CFs to iCMs was not affected by cell proliferation. Consequently, it can be concluded that CFDSV directly enhanced the conversion ratio of CFs to iCMs.

The expression patterns of relevant proteins and genes during various stages of myocardial differentiation were assessed. Immunofluorescence analysis indicated that the reprogramming of CFs into myocytes occurred relatively swiftly, with the first cTnT^+^ cells appearing by Day 3. Notably, despite the early onset of reprogramming, the progressive changes in gene and protein expression associated with cardiac muscle maturation during reprogramming persisted for several weeks. Direct evidence of this maturation was the emergence of α‐actinin as a sarcomeric structure of cardiomyocytes at 4 weeks. Tbx5, a member of the t‐box family, plays a crucial role in cardiac precursor differentiation, cardiomyocyte maturation, and the functional cardiac conduction system [36]. Western blot and RT‐PCR analyses indicated a high level of Tbx5 expression on the third day of induction, peaking at 8 days, and gradually diminishing after 12 days, suggesting that it plays a crucial role in all essential stages of myocardial development, spanning from the early naive state to the fully mature stage. Cx43, the predominant connexin in myocardial tissue, primarily present in working myocardium, facilitates intercellular information transmission, electrophysiological stability, and plays a vital role in early heart development and mediating the mechanical adhesion of mature cardiomyocytes [37, 38]. Cx43 expression increased progressively during the induction process, signifying the emergence of specialized structures for information transmission among cardiomyocytes as they formed, laying the foundation for rhythmic contraction and potential cardiomyocyte formation. Hence, myocardial‐associated proteins exhibit time‐dependent expression during the differentiation of fibroblasts into iCMs, regulating various stages of differentiation. The process progresses directly from the precursor stage to the mature stage without reverting to a cardiac progenitor cell state, potentially explaining the swift early reprogramming process. This conclusion is supported by the absence of Nkx2‐5 activation during reprogramming, which would have identified any cells transiently expressing Nkx2‐5.

This study also explored the key genes and their mechanisms during myocardial differentiation induced by small molecule compounds, accurately identifying DEGs at the gene level. Based on the gene sequencing outcomes and analysis of differential gene expression before and after induction, known marker genes of CFs and cardiomyocytes were confirmed. Gene Ontology function and KEGG pathway analyses revealed a significant upregulation of genes closely linked to myocardial development, primarily concentrated in pathways regulating cardiac muscle contraction, cGMP‐PKG signaling, cAMP signaling, calcium signaling, and rap1 signaling. Conversely, genes associated with tissue fibrosis processes exhibited a notable downregulation, primarily focused on components like elastic fibers, extracellular matrix, sarcomere structures, myofibril composition, and other constituents related to cytokine receptor, cancer, fibrosis, and the TGF‐β pathway.

In this study, the cardiac reprogramming process is enhanced by small‐molecule compounds in a time‐dependent manner and these data indicated that the CFDSV‐induced direct conversion of CFs to cardiomyocyte‐like cells rapidly and efficiently. These cells exhibited relevant markers of mature myocardium, possessed the fundamental sarcomere structure, and showed signals of Ca^2+^ flow activity. However, in comparison with cardiomyocytes, they are not fully mature as evidenced by the absence of expression of certain contractile‐specific genes, such as troponin I and N2B, and their structural and electrophysiological immaturity. For example, cardiomyocyte‐like cells display a fusiform shape, predominantly mononuclear and a few are binuclear, with an incomplete sarcomere structure. In addition, Ca^2+^ peak time is longer and Ca^2+^ signal attenuation is slower compared with mature cardiomyocytes. These suggest that full maturation is a slow process that requires complex regulation of multiple signaling networks, and further refinement of methods is necessary to enhance the promotion of maturation.

In conclusion, our findings advocate for small‐molecule therapy as an optimized strategy to enhance cardiac cell reprogramming. Given the diverse effects of chemical inducers on signaling pathways and gene transcription, further research is necessary to elucidate the precise molecular mechanisms of reprogramming. In addition, the effects of small molecule compounds on cardiomyocyte function and safety *in vivo* need to be further clarified.

## Conflict of interest

The authors declare no conflict of interest.

### Peer review

The peer review history for this article is available at https://www.webofscience.com/api/gateway/wos/peer‐review/10.1002/2211‐5463.13811.

## Author contributions

ZG and DC conceptualized this research study, validated the methodologies, performed the investigations, wrote the original draft and the final draft, and prepared visualizations. Formal analyses were done by CS and XT. HL contributed to the methodology and software. This work was administered and supervised by ZG who along with YJ performed the data validation. All authors have read and agreed to the final published work.

## Supporting information


**Table S1.** The primer sequences of related genes used for RT‐PCR.

**Table S2.** Significantly upregulated gene expression after CFDSV induction (FPKM).

**Table S3.** Significantly downregulated gene expression after CFDSV induction (FPKM).

**Movie S1.** Fluorescent image of control group.
**Movie S2.** Fluorescent image of CFDSV group.
**Movie S3.** Ca^2+^ transfer between adjacent cells was monitored by fluorescence image.

## Data Availability

The data that support the findings of this study are available from the corresponding author upon reasonable request.
