# Supplementary material for: Regulation of cardiac fibroblasts reprogramming into cardiomyocyte‐like cells with a cocktail of small molecule compounds
Source: FEBS Open Bio. 2024 May 1;14(6):983–1000. doi: 10.1002/2211-5463.13811 (PMC11148126; doi:10.1002/2211-5463.13811)
Supplement: Supplementary file 1 — Table S1. The primer sequences of related genes used for RT‐PCR. [file FEB4-14-983-s001.docx]

**Table S1**

**The primer sequences of related genes used for RT-PCR.**

| Gene | F (5'→3') | R (5'→3') |
| --- | --- | --- |
| *α-actinin* | TCCAGCCCTCTTTCATTGGT | TGCCTCCAGACAGGACATTG |
| *Tbx5* | CCCAGCAAGTCTCCATCCTC | TCATTTCTGTGCCCACTTCG |
| *Gja1* | GGTGGGCACAGACACGAATAT | CTCAACAACCTGGCTGCGAAA |
| *Tnnt2* | AAGATCAGCTGAGGGAGAAG | GGTCTTGGAGACTTTCTGGT |
| *Sox2* | CAGCTCGCAGACCTACATGA | CCTCGGACTTGACCACAGAC |
| *Tpm1* | TCACTGCAAAAGAAACTCAAGG | GTCTGTTCAGAGATGCTACGTC |
| *Tpm2* | GAAGGATGAGGAGAAGATGGAG | GTATTTGCGGTCTGAGTCCTC |
| *Myh6* | TCAGTCAGGCCAATAGAATAGC | TTCTCCTTCAGGTCGTCATTG |
| *Tgfbr3* | CCAGTCCAATTCCTCCTCCTCCTC | CTTGCTGCCTTCGTGCTGTCTC |
| *Fbln1* | TCGCTCTCCTACACCTCAGAATCC | CACAGTGCAGCTCCTCCAGTTG |
| *Col4a4* | TCCAGGCGTGGATGGTGTACC | GTGAGCCATTGTAGCCGTCCATAC |
| *Wnt5a* | AGGCGAGCTGTCTACCTGTGG | AATTCCTTGGCGAAGCGGTAGC |
| *Col7a1* | CATCGTGAGAGTGGCTTGGAACC | AGTAGGAACCACAGTCGCAGGAG |
| *Tgfbi* | ACGGTGTGGTGTATGCCATCAAC | TCATCCTCTCCGCTTCATCCTCTC |
| *Wnt2b* | CTGCTGCTGCTTCTGACTCTGC | CGGAACTGGTGTTGACACTCTCG |
| *Gapdh* | CAAGGTCATCCATGACAACTTTG | GTCCACCACCCTGTTGCTGTAG |
