# Supplementary material for: Regulation of cardiac fibroblasts reprogramming into cardiomyocyte‐like cells with a cocktail of small molecule compounds
Source: FEBS Open Bio. 2024 May 1;14(6):983–1000. doi: 10.1002/2211-5463.13811 (PMC11148126; doi:10.1002/2211-5463.13811)
Supplement: Supplementary file 2 — Table S2. Significantly upregulated gene expression after CFDSV induction (FPKM). [file FEB4-14-983-s003.docx]

**Table S2**

**Significantly upregulated gene expression after CFDSV induction (FPKM).**

| Gene | Control FPKM1 | ControlFPKM2 | Control FPKM3 | CFDSVFPKM1 | CFDSV FPKM2 | CFDSV FPKM2 | log2FoldChange |
| --- | --- | --- | --- | --- | --- | --- | --- |
| *Pdlim3* | 0.86 | 1.15 | 2.53 | 604.36 | 469.44 | 416.49 | 8.93655 |
| *Mef2c* | 0.36 | 0.28 | 0.93 | 2.11 | 3.24 | 5.68 | 2.80094 |
| *Nppa* | 0.2 | 0.19 | 0.2 | 144.51 | 129.07 | 116.68 | 9.37672 |
| *Tnnt2* | 1.65 | 2.06 | 5.18 | 48.13 | 68.82 | 76.29 | 4.48575 |
| *Tpm1* | 156.07 | 154.66 | 212.56 | 740.74 | 945.3 | 987.61 | 2.3507 |
| *Actc1* | 0.04 | 0.11 | 0.19 | 71.59 | 47.7 | 46.27 | 8.97167 |
| *Tpm2* | 169.72 | 197.97 | 145.84 | 2835.78 | 2248.42 | 2091.72 | 3.79811 |
| *Myh11* | 0.67 | 0.47 | 0.4 | 24.67 | 30.67 | 42.85 | 5.99727 |
| *Myocd* | 0.01 | 0.01 | 0.08 | 2.97 | 2.75 | 0.59 | 5.57449 |
| *Actn2* | 0.23 | 0.12 | 4.18 | 54.48 | 54.08 | 14.65 | 4.77541 |
| *Myh7* | 0.03 | 0.06 | 6.76 | 99.02 | 95.83 | 23.53 | 4.94263 |
| *Myh6* | 0.13 | 0.31 | 0.06 | 4.4 | 5.2 | 4.77 | 4.84732 |
