# Supplementary material for: Regulation of cardiac fibroblasts reprogramming into cardiomyocyte‐like cells with a cocktail of small molecule compounds
Source: FEBS Open Bio. 2024 May 1;14(6):983–1000. doi: 10.1002/2211-5463.13811 (PMC11148126; doi:10.1002/2211-5463.13811)
Supplement: Supplementary file 3 — Table S3. Significantly downregulated gene expression after CFDSV induction (FPKM). [file FEB4-14-983-s002.docx]

**Table S3**

**Significantly downregulated gene expression after CFDSV induction (FPKM).**

| Gene | Control FPKM1 | Control  FPKM2 | Control FPKM3 | CFDSV  FPKM1 | CFDSV FPKM2 | CFDSV FPKM2 | log2FoldChange |
| --- | --- | --- | --- | --- | --- | --- | --- |
| *Wnt2b* | 1.55 | 2.27 | 3.48 | 0.03 | 0.07 | 0.06 | -5.78407 |
| *Tgfbi* | 10.77 | 10.77 | 13.62 | 2.45 | 2.1 | 1.77 | -2.47401 |
| *Sox2* | 1.38 | 1.49 | 0.16 | 0.02 | 0.02 | 0.05 | -5.0938 |
| *Col7a1* | 4.27 | 4.49 | 0.49 | 0.11 | 0.1 | 0.07 | -5.0374 |
| *Tgfbr3* | 27.43 | 25.93 | 26.41 | 0.69 | 1.17 | 0.97 | -4.82213 |
| *Wnt5a* | 22.17 | 19.09 | 10.52 | 0.84 | 1.62 | 1.9 | -3.51259 |
| *Col4a4* | 6.52 | 6.88 | 0.68 | 0.52 | 1.15 | 0.67 | -2.59441 |
| *Tnc* | 69.14 | 74.46 | 267.90 | 28.86 | 26.77 | 31.47 | -2.79531 |
| *Mmp12* | 395.89 | 411.59 | 78.12 | 72.13 | 63.55 | 156.69 | -2.62316 |
| *Col1a1* | 3884.47 | 3479.59 | 4198.23 | 1625.61 | 1641.78 | 2060.33 | -2.39823 |
| *Fn1* | 1468.48 | 1972.21 | 1877.82 | 836.66 | 783.16 | 799.75 | -2.27682 |
| *Col3a1* | 2458.56 | 2462.37 | 2480.18 | 926.53 | 932.24 | 1448.32 | -2.08369 |
| *Fbln1* | 53.44 | 44.27 | 104.11 | 8.4 | 7.92 | 6.41 | -3.12808 |
